# Comparison of muscle activation imbalance following core stability or general exercises in nonspecific low back pain: a quasi-randomized controlled trial

**DOI:** 10.1186/s13102-020-00173-0

**Published:** 2020-04-15

**Authors:** MohammadBagher Shamsi, Maryam Mirzaei, Mohammad HamediRad

**Affiliations:** 1 School of Allied Medical Sciences, Kermanshah University of Medical Sciences, Kermanshah, Iran; 2 University of Illinois at Urbana-Champaign, Urbana, IL 61801 USA

**Keywords:** Core stability exercise, Electromyography, General exercise, Muscle activation imbalance, Nonspecific low Back pain

## Abstract

**Background:**

Low back pain causes changes in muscle activation patterns. Knowing how different exercises may improve altered muscle activation is useful in the treatment of patients. The aim of the study was to investigate whether there was a difference in the pattern of muscle activation in chronic nonspecific low back pain sufferers following core stability exercise (CSE) and general exercise (GE).

**Methods:**

Fifty-six non-specific chronic LBP subjects were randomly assigned to either groups (28 participants in CSE and 28 in GE group). Both groups performed 16 sessions of an exercise program for about 5 weeks. Pain, disability and trunk muscle activation patterns (using surface electromyography) were measured at baseline and post-training.

**Results:**

After the intervention period, antagonist coactivation ratio did not change in either groups. Though all compensated imbalance ratios (residual unequal muscular activity after cancellation of directionality) decreased towards negative (imbalance to left side) only this change for total muscles ratio in GE was significant (mean difference in GE group, 0.15; 95% CI: 0.02 to 0.28; *p*-value of paired t-test: 0.022); (mean difference in CSE, 0.02; 95% CI: − 0.07 to 0.11; *p*-value of paired t-test: 0.614).. No overall significantly decrease in uncompensated imbalance ratio (absolute imbalance values without cancellation directionality) was observed. Pain and disability decreased significantly in both groups. However, there was no difference between two groups in either of the variables after the intervention.

**Conclusions:**

Both exercise programs reduced pain and disability and made or kept trunk muscle activation imbalance to the left side. The effects of two exercises on pain, disability and antagonist coactivation or imbalance ratios were not different.

**Trial registration:**

This study was registered in the Iranian Clinical Trial Center with the code IRCT201111098035N1, Registered Jan 21, 2013.

## Background

Most clinical practice guidelines endorse exercise for the treatment of chronic low back pain (CLBP) including core stability exercises (CSE) and general exercises (GE) [1, 2]. Since spinal stabilization and control is altered in LBP patients [3], CSE is suggested as a treatment in recent years. These exercises aim to re-educate coactivation patterns of local and global back muscles [4]. The base of CSE is initial low-level isometric contraction of trunk stabilizing muscles (i.e. multifidus, transversus abdominis, and internal oblique) that is their integration into functional tasks progressively [4]. Good evidence exists regarding benefits of exercise (generally) in CLBP [2, 5]. In the 1990s, general strengthening exercises were more popular than other types of exercise for patients with CLBP [6].

It remains, however, controversial whether CSE is more effective than its counterparts such as GE [7]. A number of studies have suggested that CSE is more useful than other sorts of therapy for CLBP [7] while others have indicated that both exercises are equally effective [7–9]. The latter studies suggest that improvements resulted from CSE are simply due to their physiological impacts that all exercises have on patients rather than on spinal stabilization [7].

It has been claimed that LBP leads alterations in muscle activity around the location of pain [10]. So pattern of trunk muscle activation in patients with mechanical LBP (which the pain arises from structures of the spine including bones, ligaments, discs, joints, nerves and meninges [11]) is different from healthy population [12, 13]. Most authors think that the changes in muscle activity in patients with LBP should be regarded as functional adaptations to a reduced spinal stabilization [13]. Panjabi [14] first proposed that instability of the spine is likely due to any dysfunction of either spinal passive (non contractile) or active (trunk muscles) structures or from reduced neural control over these two parts and the instability could lead to LBP. Instability of the spine could cause excessive tissue strain and result in pain. Panjabi believed that to compensate a loss of passive stabilization, trunk muscles should be actively contracted. It has been shown, that co-contraction of muscles increases the stability of trunk [13]. In addition, healthy subjects when confronted with conditions that threaten spinal stability, increase co-contraction of their muscles [15].

Hodges [16] believes that dynamic control of the spine involves a spectrum of control strategies that ranges from co-contraction stiffening in one end (that is contraction of large flexor and extensor muscles causing restricted movements and high load on the spine) to more dynamic control strategies in the other (that the control of spine is achieved by timed alternating activities of global muscles with underlying tonic and early activity of deep muscles, such as the control of trunk during arm movements).

It may be useful to find how different exercises affect trunk muscles activation pattern and imbalance ratios in LBP sufferers. As CSE and GE are expected to have positive effects on these patients and they are usually used in clinics, the present investigation that is a quasi-randomised controlled trial study aims to compare them for the first time. We hypothesize that both exercise programs due to their physiological effects would make useful changes on trunk muscle activation pattern and imbalance. Patient’s disability and pain intensity were also measured and compared before and after the training.

Based on the evidence, it remains unclear whether one treatment is more beneficial than the other, so examining the differential effects of CSE and GE was our specific interest. The aim of this study was to compare the effects of CSE (The CSE group served as a treatment group) and GE (The GE group served as a control group) on trunk muscle activation patterns and imbalance in non-specific CLBP patients.

## Methods

### Study design

A quasi-randomised controlled trial was conducted. From the ethics committee of Iran University of Medical Sciences (IUMS), approval for the research was received. This study was registered in the Iranian Clinical Trial Center with the code IRCT201111098035N1, https://en.irct.ir/trial/8471, Registered Jan 21, 2013.

### Participants

Labelling participants as non-specific CLBP was based on imaging and clinical examination (pain provocation tests) by just one examiner. Fifty-six non-specific chronic LBP subjects referring to a hospital outpatient physiotherapy department volunteered (Forty-six patients completed the program). Participant characteristics are presented in Table 1. Inclusion criteria were having LBP for more than 3 months, pain intensity from 3 to 6 in visual analogue scale (VAS), and age of 18 to 60 years. Exclusion criteria were defined as history of having pathology or anomaly in lower limbs or back such as malignancy, inflammatory diseases, sever osteoporosis, arthritis or bone diseases. When admitted, patients were allocated a number in the order that they participatedthe study. Those with odd numbers were assigned to the CSE group while those with even ones to the GE group. The study was explained for all the participants at the first session and their written informed consent was obtained. Participants with a history of 3 consecutive or 5 total absences from exercise sessions were excluded.

**Table 1:** Participants Characteristics

Characteristic	Core Stability Exercise Group	General Exercise Group
Gender		
Male	11	7
Female	16	17
Age/mean (SD)	38.9 (12.2)	47.0 (9.9)
Height (cm)/mean (SD)	167.6 (8.8)	164.0 (9.1)
Weight (kg)/mean (SD)	71.9 (14.2)	74.2 (10.7)

### Interventions

Both groups performed a warm-up period (8 stretching exercises and stationary cycling for 5 min) at the beginning of every session. An eight-step exercise in which the level of difficulty increased progressively was prescribed for each group [17]. Exercises commenced with simple movements and progressed to more difficult exercises, e.g., on a Swiss ball. For the interventions to be comparable, an attempt was made for exercises to be in the similar manner for both groups in each stage. The frequency of exercise for both groups was 3 sessions per week, a total of 16 sessions. The patients were instructed to perform their exercises as much as they could. In the same session there was rest periods between the exercises. However, the net exercise time was defined to be 20 min for the CSE and 14 for the GE group (total of 320 and 224 min respectively). To balance estimated total trunk muscle force output between groups, based on previous studies [17], these times were chosen. Participants in both groups performed the exercises in the defined time duration under supervision of an experienced physiotherapist. They were blinded about the existence of two treatment groups and the exercise type they were performing. Both exercises are explained in a previous article [18] (*The exercises are shown in the* Supplementary Material File*)*.

### Core stability exercise

In this group, anatomy and function of deep lumbar stabilizer muscles were explained for patients. Recognition of these muscles’ contraction was taught in the first sessions*.* To ensure accurate contraction of the transversus abdominis muscle, it was explained to the patients that by the action of this muscle the lower part of the anterior abdominal wall below the umbilical level will be “drawn in”. To be sure of multifidus contraction, bulging action of the muscle was felt under the therapist’s fingers when they were placed on either side of the spinous processes of lumbar vertebrae (directly over the belly of this muscle) [4]. Then, low-intensity isometric contraction of these muscles in minimally loading positions was prescribed. Step by step, integration with dynamic activities was instructed. This was done by performing light functional tasks while performing co-contraction of the stabilizing muscles. In the 6 last sessions of the program, heavier-load functional tasks with exercises similar to those in the GE group were progressively instructed.

### General exercise

For the GE group, exercises were conducted which activated the extensor (paraspinal) and flexor (abdominal) muscles. The participants performed these exercises in lying position.

### Outcome measures

Three variables were measured before and after the intervention for each participant including: 1- Disability and Pain 2- Trunk muscle activation patterns (Electromyographic Ratios).

#### Disability and pain

To measure the degree of disability and to estimate their quality of life, all participants completed the Persian translated version of Oswestry Disability Questionnaire [19] (0 = no disability, 100 = totally disabled), and their pain intensity was assessed using visual analogue scale (VAS) (0 = no pain, 100 = pain as bad as it could be).

### Trunk muscle activation patterns (Electromyographic ratios)

#### EMG recording

Surface electromyographic signals were collected using ME6000 device (MEGA Electronics Ltd., Kuopio, Finland) with surface electrodes (Ag/AgCl). An inter-electrode distance of 2 cm was maintained. CMRR was 110 dB. After the skin was abraded and cleaned with alcohol (to reduce skin impedance until it was lower than 5 k Ω), the electrodes were placed bilaterally (right,left) over the following trunk muscles and locations [20]: rectus abdominis, approximately 3 cm lateral to the umbilicus; external oblique, approximately 10 cm lateral to midline above the umbilicus and aligned with muscle fibers; internal oblique, 2 cm below and 7 cm medial to the anterior superior iliac spine; longissimus dorsi, approximately 3 cm lateral to midline at the L1; iliocostalis approximately 6 cm lateral to midline at the L3. The EMG signals were sampled at 10,000 Hz and band passed between 20 and 450 Hz, full-wave rectified low pass filtered (second order single pass Butterworth) at a cut-off frequency of 2.5 Hz. The last 1 sec of EMG signal while subjects were in the proper test condition was ignored and previous 3 sec were selected and averaged for analysis. To assess the reliability of the EMG signals, 12 healthy subjects were tested two times in the same manner as the patients.

##### Tasks

**Fig. 1:**
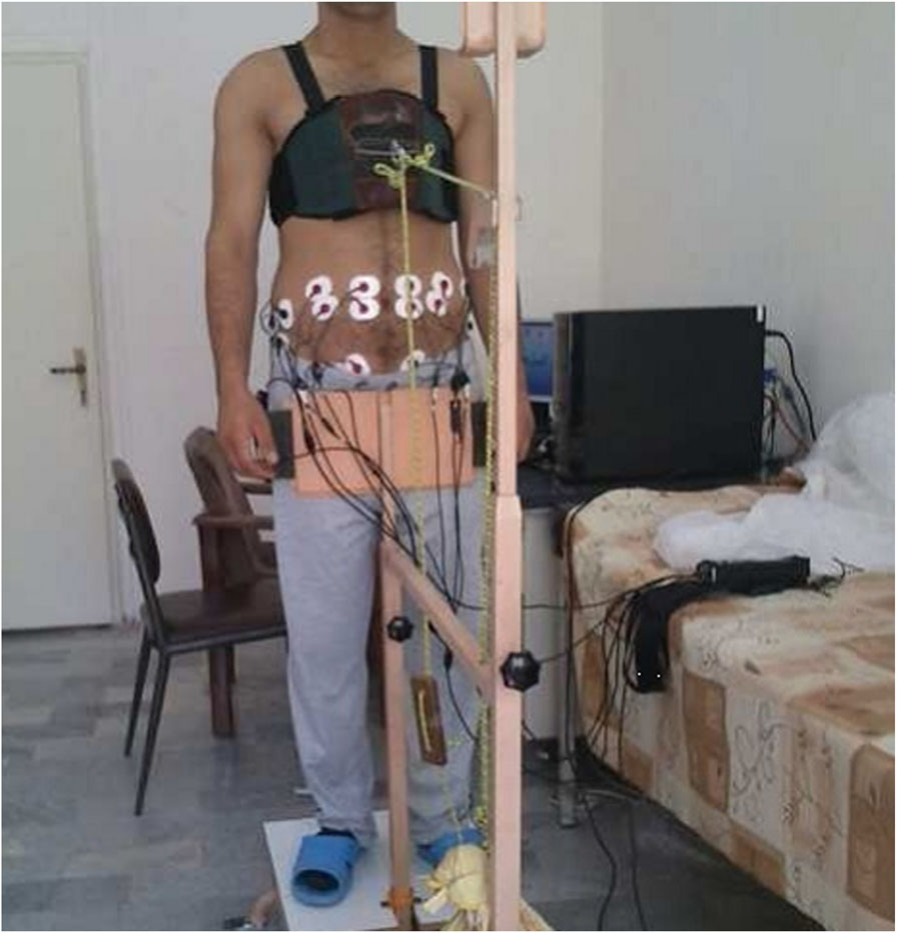
Forward pull position

**Fig. 2:**
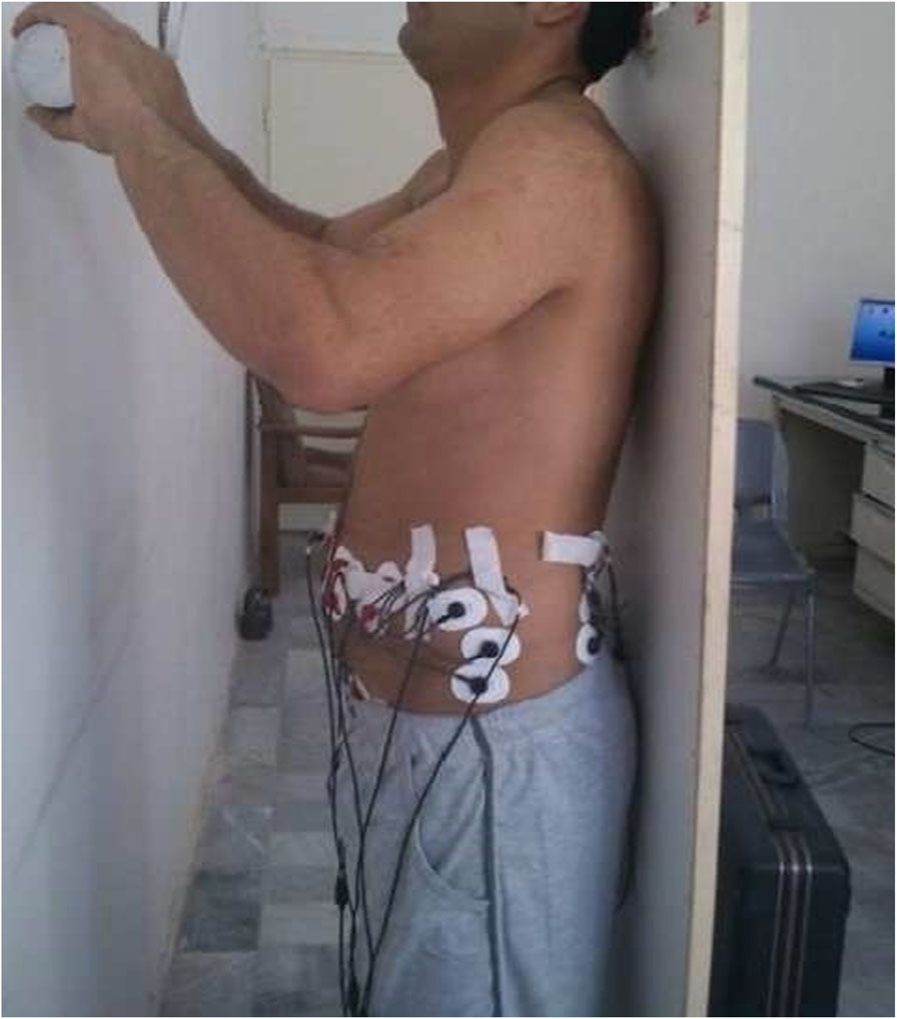
20, 40 and 60 cm holding weight position

EMG signal acquisition was conducted in 5 positions in two different conditions challenging trunk stability:

1. Forward and backward pull positions (FPP & BPP): The participants were placed in a standing position on an apparatus and strapped into a postural restraint that restricted their hip and lower limb motion but left their upper torso free to move in any direction. They wore a harness having a hook for applying a torque to the trunk by a rope (Fig. 1). Regardless of their different trunk height, based on the distance between the hook and the L_5_ –S_1_ interspace, the amount of weight pulling the trunk by the rope was changed so that a constant torque of 40 N.M. was applied for all participants. Subjects were placed on the apparatus in two positions. Their trunk were pulled in forward and backward directions [Forward (Fig. 1) and backward (the subject turn 180 degrees around regarding Fig. 1)] pull positions. They held these conditions for 5 s.
2. Holding weight when standing: The participants were placed upright with their toes 36 cm apart from a wall. They were instructed to hold a 4.5 kg dumbbell by their hands at the three heights of 20, 40 and 60 cm above their fifth lumbar spine which were marked on the wall. They were asked to keep the weight in the distance of about 1–2 cm from the wall while keeping their upright posture (Fig. 2). They held this condition for 5 s.

##### Electromyographic ratios

To assess trunk muscle activation patterns and imbalance in participants performing the tasks, two types of ratio were calculated including:

1. Antagonist Coactivation (AntC) Ratio: Root mean squares (RMS) of EMG signals for all muscles were calculated firstly. Simple sum of RMS for antagonists over sum of RMS for all muscles was computed., For the “forward pull position” and also for the “holding weight in standing positions (20, 40 and 60 cm)” the sum of values for rectus abdominis and oblique muscles, and for the “backward pull position” the sum of values for back muscles, were divided by the sum of values for all muscles.
2. Imbalance ratios: Using analysis similar to Oddsson and DeLuca [10], to assess imbalance in trunk muscles three types of ratio were calculated:

A- Extensor Ratio: Using longissimus dorsi and iliocostalis muscles.
B- Flexor Ratio: Using rectus abdominis, external oblique, and internal oblique muscles.
C- Total Ratio: Using all back and abdominal muscles.

The RMS signals from pair of those five muscles were used. For each muscle group (A, B or C), the right-side RMS values were divided by the left-side. Then, using the following procedure (Eq. 1), each of the ratio values was transformed to make corrected ratios (R) which for symmetrical properties are centered on 0. To show the difference in percent between the right and left sides, the obtained ratio was multiplied by 100.

1 $$ R=\left\{\begin{array}{cc}\mathrm{ratio}-1,& \mathrm{ratio}\ge 1\\ {}-\left(\frac{1}{\mathrm{ratio}}-1\right),& \mathrm{ratio}<1\end{array}\right. $$

For example, the meaning of a value of − 10 is that the left side was 10% larger than the right side whereas the meaning of a value of 10 is that the right side was 10% larger than the left side. It is possible to make comparison between right- and left-sided differences in EMG signals by these ratios [10].

Using the procedure which is shown in the eqs. 2 and 3, two other EMG measures were then calculated from each muscle ratio parameters. “Uncompensated” imbalance was described as the mean across “absolute” values of all single muscle ratios in each muscle group (Extensor, Flexor and Total) (Eq. 2). For example, uncompensated imbalance extensor ratio was calculated by averaging absolute ratio values for longissimus dorsi and iliocostalis muscles. “Compensated” imbalance was described as the mean across (not absolute) values of all single muscle ratios in each muscle group (Eq. 3). So, six values were calculated for each participant: extensor uncompensated (ExtUC) and compensated (ExtC) imbalance, flexor uncompensated (FlxUC) and compensated (FlxC) imbalance and total uncompensated (TotUC) and compensated (TotC) imbalance ratios.

2 $$ \mathrm{Uncompensated}\ \mathrm{Imbalance}=\mid {\mathrm{ratio}}_{\mathrm{Muscle}1}\mid +\mid {\mathrm{ratio}}_{\mathrm{Muscle}\ 2}\mid +\mid {\mathrm{ratio}}_{\mathrm{Muscle}\ \mathrm{n}}\mid /\mathrm{n} $$

3 $$ \mathrm{Compensated}\ \mathrm{Imbalance}={\mathrm{ratio}}_{\mathrm{Muscle}1}+{\mathrm{ratio}}_{\mathrm{Muscle}\ 2}+{\mathrm{ratio}}_{\mathrm{Muscle}\ \mathrm{n}}/\mathrm{n} $$

(Muscle 1, 2 and muscle n are muscles that have been defined in a muscle group (A, B or C) and n is the number of muscles in the group).

The uncompensated imbalance ratio is an index showing the total muscular imbalances regardless of either to right or left, whereas the compensated imbalance shows the direction of the local segmental imbalances. Therefore a positive value indicates that right is larger than left and a negative value shows the opposite.

Also in compensated imbalance ratios, there may be cancellation between the different muscles within each subject so they represent the residual imbalance. In order to avoid cancellation of values of compensated imbalances with opposite signs, absolute values of the compensated imbalances were used.

To have a better insight about each variable, for each participant, mean values for all five positions (FPP, BPP, 20, 40 and 60 cm) were calculated and defined as a new variable (Mean). So the number of ratios calculated for five positions reduced to one mean value.

### Statistical analysis

The normality of the data was confirmed using the K–S test. Independent t-test and Chi-square test were used to examine differences between the two study groups in demographic characteristics and baseline values of disability level, pain intensity and EMG ratios. Analysis of covariance (ANCOVA) was used to test the significance of changes in scores of muscle coactivation and their imbalance ratios (ExtUC, ExtC, FlxUC, FlxC, TotUC, TotC) between the two groups, controlling the baseline values. Within-group changes before and after the study were assessed by paired t-test. The intra-class correlation coefficient (ICC) and standard error of measurements (SEM) were used to assess the relative and absolute reliability of EMG signals, respectively.

## Results

**Fig. 3:**
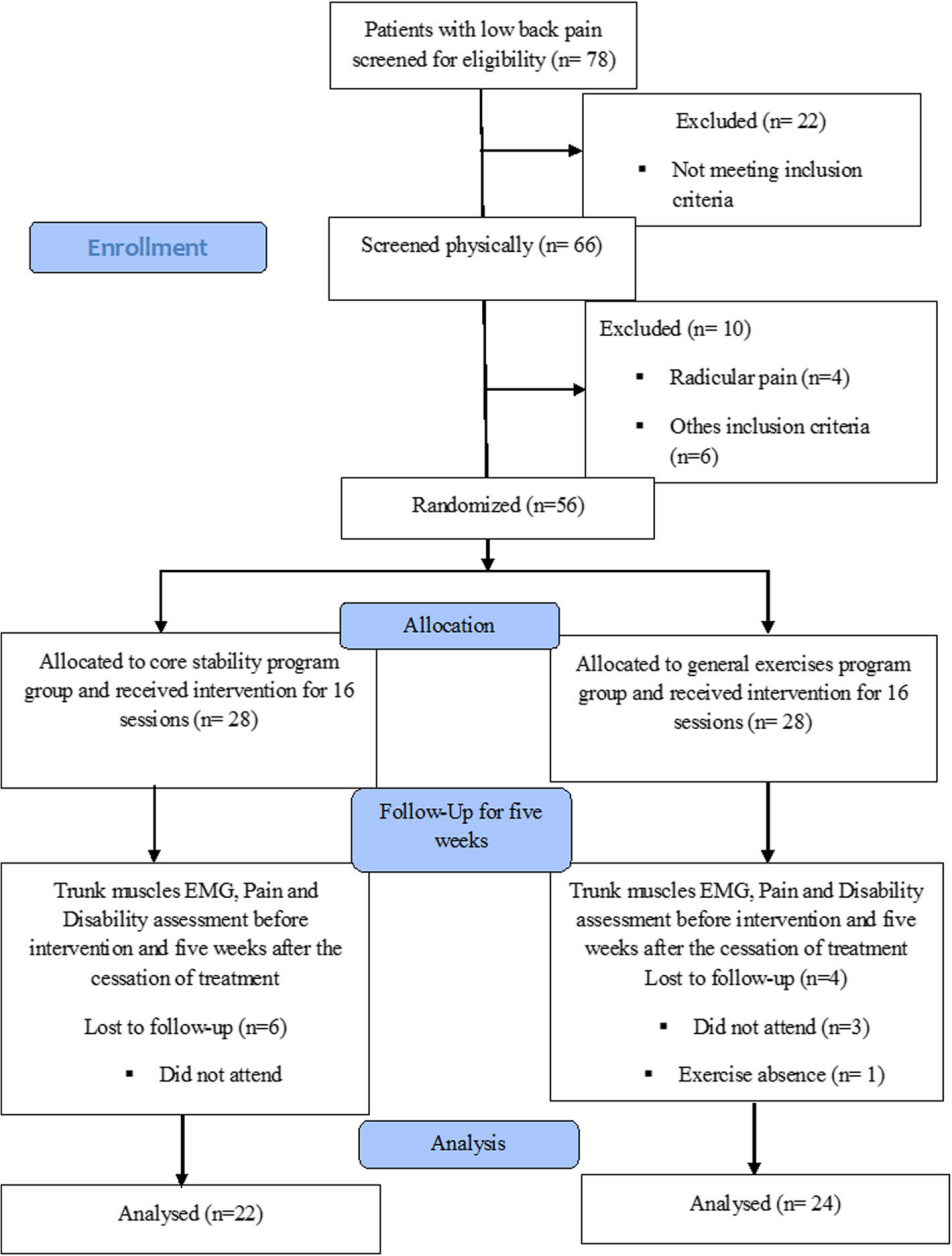
Flow diagram of participation in the study

Different phases of the trial are presented in the Fig. 3. In our study, we had ten dropouts out of 56 participants fulfilling inclusion criteria and 46 participants remained (22 participants in CSE and 24 in GE group). ICC and SEM for EMG values for all muscles in different positions ranged over 0.66 to 0.99 and 0.001 to 0.10, respectively. The relative measure of reliability was good (ICC > 0.90) and absolute measure of reliability (SEM) showed low values.

There was no significant statistical difference in disability (*p* = 0.91) and pain (*p* = 0.23) between groups on entry to the trial. After the intervention period, a significant reduction in disability level (*p* < 0.001) and pain intensity (*p* < 0.001) within each group was found (Table 2). Regarding changes in outcomes (the difference between before and after treatment values), there was no significant difference between CSE and GE groups in disability (*p* = 0.14) and pain (*p* = 0.72) (Table 2).

**Table 2:** Means, Standard Deviations (SD), and Within and Between Group Differences for Disability and Pain in the Exercise Groups Following the Intervention Period

	Core Stability Group			General Exercise Group			
Outcome Measures	Before	After	*P*-value for Difference^a^	Before	After	*P*-value for Difference^a^	*P*-value for Difference Between Groups^b^
Oswestry disability	50.55 (12.08)	32.77 (11.0)	*P* < 0.001	50.67 (10.41)	37.62 (10.87)	*P* < 0.001	*P* = 0.14
Pain intensity	51.36 (9.02)	15.09 (12.4)	*P* < 0.001	52.86 (9.02)	15.10 (13.80)	*P* < 0.001	*P* = 0.72

^a^*P*-value for paired t test

^b^*P*-value for ANCOVA, adjusted for baseline values

There was no significant statistical difference in Antagonist Coactivation (AntC) Ratio (*p* = 0.08 to *p* = 0.47) and imbalance (ExtUC, ExtC, FlxUC, FlxC, TotUC, TotC) ratios (from *p* = 0.06 to *p* = 0.99) between groups on entry to the trial. The mean values for antagonist coactivation, compensated and uncompensated RMS imbalance ratios are shown in Table 3.

**Table 3:** Means, Standard Deviations (SD), and Within and Between Group Differences for Muscles Coactivation Pattern and their Imbalance Ratios in the Exercise Groups Following the Intervention Period

Variable	Baseline values		After 5 weeks of intervention		*P*-value^a^	*P*-value^a^	*P*-value^b^
	CSE group	GE group	CSE group	GE group	(Differences within CSG)	(Differences within GEG)	(Differences between groups)
AntC	0.38 (0.18)	0.32 (0.14)	0.36 (0.14)	0.28 (0.08)	0.428	0.065	0.647
ExtUC	0.45 (0.68)	0.51 (0.87)	0.27 (0.03)	0.40 (0.23)	0.220	0.577	0.830
ExtC	−0.015 (0.45)	0.096 (0.69)	−0.094 (0.24)	− 0.16 (0.30)	0.418	0.801	0.402
FlxUC	0.433 (0.54)	0.29 (0.16)	0.35 (0.21)	0.27 (0.13)	0.347	0.457	0.779
FlxC	−0.059 (0.29)	0.054 (0.25)	−0.042 (0.23)	−0.032 (0.19)	0.710	0.102	0.118
TotUC	0.442 (0.57)	0.378 (0.34)	0.32 (0.139)	0.319 (0.11)	0.243	0.458	0.590
TotC	−0.042 (0.17)	0.071 (0.30)	0.06 (0.19)	−0.084 (0.16)	0.614	0.022	0.096

*AntC* Antagonist Coactivation, *ExtUC* extensor uncompensated extensor compensated, *ExtC* extensor compensated, *FlxUC* Flexor uncompensated, *FlxC* Flexor compensated, *TotUC* total uncompensated, *TotC* total compensated imbalance ratios, *CSE* core stability exercise, *GE* general exercise

^a^*P*-value for paired t test

^b^*P*-value for ANCOVA, adjusted for baseline values

After 16 sessions of intervention, AntC ratio did not change in either groups ((mean difference in GE group = 0.02; 95% CI: − 0.03 to 0.08; *p*-value of paired t-test: 0.428) and (mean difference in CSE group = 0.05; 95% CI: 0.001 to 0.09; *p*-value of paired t-test: 0.068)). The mean TotC ratio showed a significant decrease in GE group (mean difference, 0.15; 95% CI: 0.02 to 0.28; *p*-value of paired t-test: 0.022) while the CSE group showed no significant changes for TotC ratio (mean difference, 0.02; 95% CI: − 0.07 to 0.11; *p*-value of paired t-test: 0.614). Furthermore, ExtUC, ExtC, FlxUC, FlxC and TotUC were not significantly different compared to baseline in both group (All *P* > 0.05) (Table 3).

In between-group analysis, no significant difference was observed for muscle coactivation pattern and their imbalance ratios at the end of the study (all *P*-values > 0.05; Table 3).

## Discussion

The current study compared trunk muscles coactivation and imbalance patterns between two groups of patients with chronic non-specific LBP enrolled in two types of exercise program. At the end of the study, antagonist coactivation did not reduced in either groups. Sixteen sessions of training in both groups shifted compensated ratios (residual unequal muscular activity after cancelation of directionality) (ExtC, FlxC and TotC) to negative, indicating change in muscle imbalance to the left side. The only significant change was TotC for the general exercise group.

*In a survey on the literature,* no study was found on comparison between core stability and general exercises regarding recruitment pattern and activation imbalance. Trunk muscle imbalance in LBP compared with healthy subjects has been reported in some studies [10, 21] though other studies [22] have failed to show differences in muscle activity between these two groups.

We measured EMG for global (and not local) muscles, and decrease in antagonist coactivation in GE group could be attributed to the change from static to dynamic spinal control whereas this change has not occurred in CSE group which is claimed [23] to cause enhancement in spinal stability specifically.

Regarding imbalance in trunk muscles, Oddsson and Carlo [10] found similar levels of uncompensated imbalance in LBP patients and healthy control participants whereas they found that high compensated RMS imbalance, i.e., large residual activation imbalances, was a sign of unhealthy back muscle function in LBP patients. The concept of uncompensated and compensated EMG-based imbalance parameters has been introduced to show how contralateral muscles in the trunk are contracted during a sustained isometric contraction in a symmetrical task [10]. These ratios show how much load each side shares and how much work the muscles of each side do. In a similar designed work, Reeves and Cholewicki [24] had different results of equal activation imbalance between two sides for athletes with a history of low back injury and healthy athletes. They believed the differences between the studies could stem from the populations being used. For example, the LBP sufferers, unlike the athlete population, may show more pain avoidance behaviour which could cause lesser muscle activity.

The fact that the force developed by a muscle is partly proportional to the amplitude of the EMG signal [25] is the physiological rationale for the interpretation of these ratios. Uncompensated imbalances show that contralateral muscle groups do not activate equally, whereas the compensated imbalances indicate the residual unequal muscular activity after cancelation of directionality (right-left) of these imbalances. So, for each participant, a positive compensated imbalance means that values for the right side are greater than those for the left side, and vice versa for a negative value. If the uncompensated imbalance is equal to the compensated imbalance, the imbalance at all muscles will be in the same direction. When the compensated imbalance is smaller than the uncompensated imbalance, then some positive and negative values have been canceled by each other, i.e., at least one level is negative and/or one is positive.

All compensated ratios in either groups decreased, indicating imbalance shift to the left side, though the only significant change was that for the TotC ratio in GE. Therefore, both training programs made the imbalance change more or less, to the left side. Before the intervention, FlxC and TotC for the CSE group were negative and other compensated values were positive. This difference in imbalance direction at the baseline could be attributed either to the distribution of alterations in muscle activity around the location of pain or to the random chance for side dominancy, in the case of random allocation of participants in groups. The net finding is that both exercises made or kept imbalance to the left.

Though we have not asked the participants their dominant side, as most people including our subjects are right-handed [26], this change may be attributed to hand dominance, especially since our training was symmetric. Some authors [27, 28] have pointed out that dominant to non-dominant strength imbalances are normal to some extent. In a study of neuromuscular imbalance in tennis players with low back pain [29], nearly all right-handed athletes showed significant lower muscle activity on the left side of erector spinae, and left-handed players showed lower activity on the right side. In our study, the reverse non-dominant to dominant side imbalance after the intervention may be related to the effects of exercise programs for low back pain that have changed the direction of imbalance. However, it remains unclear why the muscle imbalance really shifted to the left side due to either exercises. Future studies are recommended to investigate why the muscle imbalance shifted to the left side after the exercise programs.

In spite of the general decrease in uncompensated ratios (unless FlxUn), since these changes are not significant, it could not be concluded that either exercises decreased muscle imbalance, though their trends are toward imbalance reduction.

Unlike coactivation and imbalance ratios, improvement in clinical outcomes (pain and disability index) occurred in both groups without significant difference between them. It could be interpreted that both exercises made useful effects on clinical symptoms regardless of whether they made change on muscle activation patterns. However, this question remains unanswered whether changing trunk muscle activation affects pain and other clinical features.

However, the findings of this study could be used in motor control studies which investigate the behaviour of trunk muscles of patients suffering from LBP after a course of therapeutic exercise.

### Limitations

The main limitations of this study are the lack of a true control group in the design and performance and lack of blindness for the treating physiotherapist due to the nature of the interventions. Having a control group in the future studies would be useful.

Despite the limitations, we believe the results of the current study can add to the literature that there are no significant differences in pain, muscle activation patterns and imbalance ratios of trunk muscles between LBP patients who engaged in CSE group versus those engaged in GE group.

The participants were not randomly allocated to the study arms, which can be considered as a drawback of our study. Minimize systematic bias or confounding could not be achieved using the quasi-randomized trial design.

## Conclusion

Though both interventions caused a decrease trend on antagonist coactivation, but they were not significant. All muscle imbalance ratios in either groups shifted to the left side, though the only significant change was that for the TotC (compensated all back and abdominal muscles) ratio only in general exercise group. Pain and disability reduced in both CSE and GE programs. The effects of two exercises on pain, disability and antagonist coactivation or imbalance ratios were not different.

## Data Availability

The datasets used and/or analysed during the current study are available from the corresponding author on reasonable request.

## Abbreviations

AntC: Antagonist Coactivation
CLBP: Chronic low back pain
CSE: Core stability exercises
EMG: Electromyographic
GE: General exercises
ICC: Intra-class correlation coefficient
IUMS: Iran University of Medical Sciences
RMS: Root mean squares
VAS: Visual analogue scale
